# Ferroptosis and iron-based therapies in *Pseudomonas aeruginosa* infections: From pathogenesis to treatment

**DOI:** 10.1080/21505594.2025.2553787

**Published:** 2025-09-05

**Authors:** Dong-Han Shao, Yu-Feng Yao, Dan-Ni Wang

**Affiliations:** a Shanghai Institute of Immunology, Shanghai Jiao Tong University School of Medicine, Shanghai, China; b Laboratory of Bacterial Pathogenesis, Department of Microbiology and Immunology, Institutes of Medical Sciences, Shanghai Jiao Tong University School of Medicine, Shanghai, China; c Department of Infectious Diseases, Shanghai Ruijin Hospital, Shanghai, China; d State Key Laboratory of Microbial Metabolism, and School of Life Sciences and Biotechnology, Shanghai Jiao Tong University, Shanghai, China; e Shanghai Key Laboratory of Emergency Prevention, Diagnosis and Treatment of Respiratory Infectious Diseases, Shanghai, China

**Keywords:** *P. aeruginosa*, iron acquisition, iron metabolism, ferroptosis, drug-resistance

## Abstract

*Pseudomonas aeruginosa* is a globally prevalent multidrug-resistant pathogen that causes severe infections, particularly in immunocompromised individuals. This review focuses on the dual role of iron in *P. aeruginosa* infections: as a critical nutrient for bacterial growth and as a mediator of host cell ferroptosis, a form of iron-dependent cell death. We summarize how *P. aeruginosa* manipulates iron metabolism to induce ferroptosis in host cells, thereby promoting its own survival and pathogenicity. Additionally, we explore therapeutic strategies targeting iron metabolism, including interfering with acquisition of iron ions from the environment, disrupting bacterial iron metabolism and iron homeostasis, using ferroptosis inhibitors to suppress host cell ferroptosis, and employing high iron concentrations to induce bacterial ferroptosis. These insights provide innovative approaches to combat drug-resistant *P. aeruginosa* infections.

## Introduction

*Pseudomonas aeruginosa* is a common opportunistic pathogen in human which can cause pneumonia, blood stream, respiratory, urinary tract and skin infections, especially when the immune system is weakened [1–4]. It is also prone to infection in people with cystic fibrosis, leading to severe nosocomial infection [5]. Tissues infected with *P. aeruginosa* undergo various pathological changes, including inflammatory responses, cytokine secretion, immune cell infiltration, blood-air barrier dysfunction, leakage, and cell death, etc. [6]. Its pathogenic mechanisms are largely attributed to its capacity to form biofilms, quorum sensing, and metabolic adaptability and produce a wide array of virulence factors that enhance its survival in hostile environments and escape from the host immune system [7–10].

In addition, *P. aeruginosa* is highly resistant to antibiotics. *P. aeruginosa* has developed multidrug-resistant strains due to mechanisms like outer membrane permeability reduction and its component lipopolysaccharides (LPS), efflux pumps, genetic changes, and biofilm formation, posing a global health burden [11–13]. The World Health Organization identifies carbapenem-resistant *P. aeruginosa* as urgently needing new antibiotics [14]. The rise of multidrug-resistant strains has become a pressing public health concern, complicating treatment regimens and leading to increased healthcare costs and prolonged hospital stays [15]. Therefore, given the potent virulence and formidable antibiotic resistance of *P. aeruginosa*, there is an urgent need for the development of novel therapeutic strategies.

Iron metabolism plays a crucial role in the survival and pathogenicity of *P. aeruginosa* [16]. As an essential nutrient for bacterial growth, iron is often a limiting factor due to its low solubility [17]. To overcome this challenge, *P. aeruginosa* has evolved sophisticated iron acquisition mechanisms, including siderophore production and heme uptake, which enhance its adaptability and facilitate host tissue colonization [18,19]. This ability not only enhances its survival but also contributes to its virulence, making iron metabolism a potential target for therapeutic intervention. Investigating the interplay between iron availability and *P. aeruginosa* pathogenicity could lead to novel strategies for managing infections caused by this resilient pathogen.

When *P. aeruginosa* infects a host, it can induce ferroptosis in macrophages by regulating host iron metabolism, thereby evading immune clearance and promoting infection [20]. Simultaneously, iron metabolism offers unique targets for developing novel anti-infection strategies, such as interfering bacterial iron uptake and utilization, using bacterial siderophores and antibiotic conjugation to kill bacteria, inhibiting the occurrence of ferroptosis in host cells and inducing bacterial ferroptosis through high iron concentrations. This review provides a comprehensive overview of the mechanisms by which *P. aeruginosa* induces ferroptosis in host cells and explores the potential of iron-based therapeutic strategies.

## The role of iron metabolism in *P. aeruginosa*

### Mechanisms of iron acquisition and Efflux

*P. aeruginosa* acquires iron from host tissues by producing two main siderophores – pyoverdine and pyochelin. Pyoverdine is a highly efficient but metabolically expensive siderophore, which is primarily produced under severe iron limitation due to its high affinity, whereas pyochelin is synthesized when iron is only moderately restricted [21]. Pyoverdine-iron complexes are taken up by the TonB-dependent transport proteins (TBDTs) FpvA and FpvB, while pyochelin is mainly transported via FptA [22–24]. Additionally, the bacterium can utilize xenosiderophores from other microorganisms like *E. coli* and fungi, such as aerobactin, ferrichrome, ferrioxamine B, among others. This ability enhances its iron acquisition capacity, as these heterologous siderophores are transported through specific receptors like ChtA, FoxA, and FiuA [25,26]. Besides siderophores, *P. aeruginosa* can also utilize the host’s heme as an iron source. Since iron in heme accounts for the majority of iron stores in the host [27], the ability of *P. aeruginosa* to acquire iron ions from host heme provides a significant advantage. Previous studies have identified three major heme uptake systems, PhuR, HasR, and HxuA, which are located in the outer membrane and play a critical role in chronic infections, particularly in cystic fibrosis patients [28,29]. Ferrous ions can enter the cell directly through a GTP-coupled receptor, such as the Feo system, including proteins like FeoB which contains three main domains including the G-protein domain, the GDP dissociation inhibitor (GDI) domain and the transmembrane domain [30,31] (Table 1).

**Table 1.** The pathways, biological roles, regulatory mechanisms, and potential therapeutic strategies for iron acquisition in *P. aeruginosa.*

Iron Acquisition	Biological function	Regulation	Potential Therapy Strategies	Ref
Pyoverdine	A high-affinity siderophore; Interspecies competition; Biofilm formation; Bacterial drug resistance	Transcription factor AlgR; Kinase Stk1; Fur, PvdS; TonB dependent transporting protein (TBDT) FpvA and FpvB	Engineering pyoverdine analogs; Galangin; Nanoparticle; FpvB for antibiotic transport	[19,32–39]
Pyochelin	A siderophore with a smaller quantity; Survival in iron-restricted environments; Biofilm formation; Bacterial drug resistance	QS signal; sRNA like *PrrH*; Fur, PvdS, and FpvI; FptA (Pyochelin), TonB dependent transporting protein (TBDT) FptA	Competitive inhibition; QS inhibition; Biofilm formation inhibition	[19,37,38,40–46]
Xenosiderophrones	Survival in iron-restricted environments; Interspecies competition	ChtA, FoxA, FemA, and FiuA facilitate the transport of xenosiderophrones; Fur regulates expression levels	Disrupting TonB dependent transporting protein (TBDT)	[17–19,38,47–51]
Feo system(FeoB)	Transporting Ferrous Ions; Causing chronic infection patients such as those with cystic fibrosis	Iron ion concentration; GTPase-activating regulatory factors; Conserved cysteine residues	FeoB mutant can’t grow under low-iron conditions; Citrate mediated Fe^2+^ uptake may cause oxidative stress	[31,52–57]
Heme uptake system	Sequestering host heme; Biliverdin promotes biofilm formation; HemN2 participates in the synthesis of virulence factors 7-hydroxytropolone	TonB dependent transporting protein (TBDT) PhuR,HasR, HxuA; sRNA *PrrH*	Biliverdin reductase inhibition	[28,29,39,58,59–61]

However, due to the potential for high concentrations of extracellular iron ions to cause cellular membrane damage and the production of reactive oxygen species (ROS), siderophores and sources of iron are precisely regulated, and the bacterium’s iron export mechanism is also crucial [58,62]. *P. aeruginosa* avoids iron overload through ferritin and iron efflux systems, and ensures that the supply of iron meets metabolic needs while preventing toxicity by finely regulating iron acquisition and removal systems [58]. In *P. aeruginosa*, iron export through cation diffusion facilitator (CDF) system is the only way to efflux iron, *P. aeruginosa* contains three CDF metal ion transporters, one of them is alternative iron transport protein (AitP), which exports Fe^2+^/Co^2+^ while the others mediate Zn^2+^ efflux [63]. The presence of efflux pumps keeps the metabolism of iron ions within the bacterium in a steady state, which is of great significance for the survival of *P. aeruginosa*.

### Effects of iron on the growth, reproduction and pathogenicity of *P. aeruginosa*

Iron metabolism plays a crucial role in the growth, reproduction, virulence, and development of drug resistance in *P. aeruginosa*. Disruption of iron uptake and metabolism can lead to a reduction in the virulence of *P. aeruginosa*. For example, the pvdE gene, which is crucial for pyoverdine synthesis, enables pyoverdine to acquire iron ions from iron-binding proteins such as transferrin and lactoferrin [64]. Studies have shown that a *pvdE* mutant exhibits reduced virulence in ocular infections, highlighting the importance of these mechanisms [64].

It is well known that iron is a cofactor for bacterial cytochromes, reductases, and enzymes related to DNA synthesis [65]. It is involved in the electron transport chain, energy metabolism, and nucleotide synthesis and is crucial for fundamental life activities. In *P. aeruginosa*, it also plays a significant role in its virulence, antibiotic resistance, and interspecies competition. The iron it acquires can regulate Cyclic dimeric guanosine monophosphate (c-di-GMP) levels in *P. aeruginosa* by modulating the interaction between an iron-sensing protein (IsmP) and a diguanylate cyclase (ImcA). Binding of iron to the CHASE4 domain of IsmP inhibits the interaction between IsmP and ImcA, thereby increasing the synthesis of c-di-GMP by ImcA and promoting biofilm formation [66]. In patients with cystic fibrosis, *P. aeruginosa*, *Aspergillus fumigatus*, and *Candida albicans* are the predominant bacterial and fungal pathogens. *P. aeruginosa* depletes iron in the environment by restricting iron uptake by other microbes and secretes phenazine toxins to manipulate the redox and iron homeostasis of other microorganisms [67]. Additionally, studies have shown that the production of pyoverdine is directly related to cefiderocol tolerance. High pyoverdine-producing strains can provide cross-protection to susceptible *P. aeruginosa* and other Gram-negative bacteria, thereby promoting the spread of antibiotic resistance [40].

In conclusion, the interplay between iron availability and the growth dynamics of *P. aeruginosa* is crucial for understanding its pathogenesis and developing effective therapeutic strategies. Enhanced knowledge of how iron influences virulence factors, and the potential of novel therapies targeting iron metabolism, offers promising horizons in combating infections caused by this resilient bacterium.

## Ferroptosis mechanism and molecular process

### Definition and biological mechanism of ferroptosis

Ferroptosis was first proposed in 2012, and research in this field has grown exponentially [68]. Ferroptosis is an iron-dependent form of non-apoptotic cell death caused by dysregulation of intracellular iron homeostasis. This process is characterized by lipid peroxidation, inhibition of glutathione peroxidase 4 (GPX4) and system Xc^−^, resulting in the accumulation of phospholipid hydroperoxides (PLOOH) [69]. This initiates the Fenton reaction, which rapidly amplifies PLOOH. The resulting PLO• and PLOO• radicals interact with polyunsaturated fatty acyl (PUFA) moieties on phospholipids, leading to further production of PLOOH [68,70,71]. Furthermore, iron ions are involved in enzyme-catalyzed reactions related to lipid peroxidation (such as cytochrome P450 oxidoreductase); excessive iron ions entering the cell can lead to cell ferroptosis [72]. Moreover, as a distinct form of iron-dependent programmed cell death, ferroptosis has been demonstrated to generate oxidative products that regulate cellular functions and play significant roles in tumor suppression and neurodegenerative diseases [73–75].

### Mechanisms of *P. aeruginosa*-induced host ferroptosis

In recent years, numerous studies have shown that *P. aeruginosa* can suppress host immune responses and induce ferroptosis in host cells, characterized by iron-dependent lipid peroxidation and glutathione metabolism dysregulation [52,76,77]. *P. aeruginosa* can induce ferroptosis in host cells through multiple mechanisms, including production of species-specific lipoxygenases, inhibition of host GPX4 activity, and siderophore-mediated disruption of iron homeostasis. Typically, the 15-lipoxygenase in human cells initiates the ferroptosis process by acting on polyunsaturated fatty acid molecules, especially ω-6 and ω-3 fatty acids. This bacterium also synthesizes a lipoxygenase (pLoxA) with similar biological functions, which elicits pro-ferroptotic signals in host cells (e.g. 15-HpETE-PE in mouse cells) [78,79]. Iron ions are essential for the function of this lipoxygenase; only the iron-bound lipoxygenase (LOX-Fe^3+^/LOX-Fe^2+^) can catalyze the oxidation of unsaturated lipids to produce reactive oxygen species (ROS), thereby enhancing the pathogenicity of the pathogen, promoting host cell death, and leading to pathological phenomena such as tissue edema, excessive cytokine secretion, inflammatory cell infiltration, and hypoxia [20,52,78,80] (Figure 1(a)).

**Figure 1.**
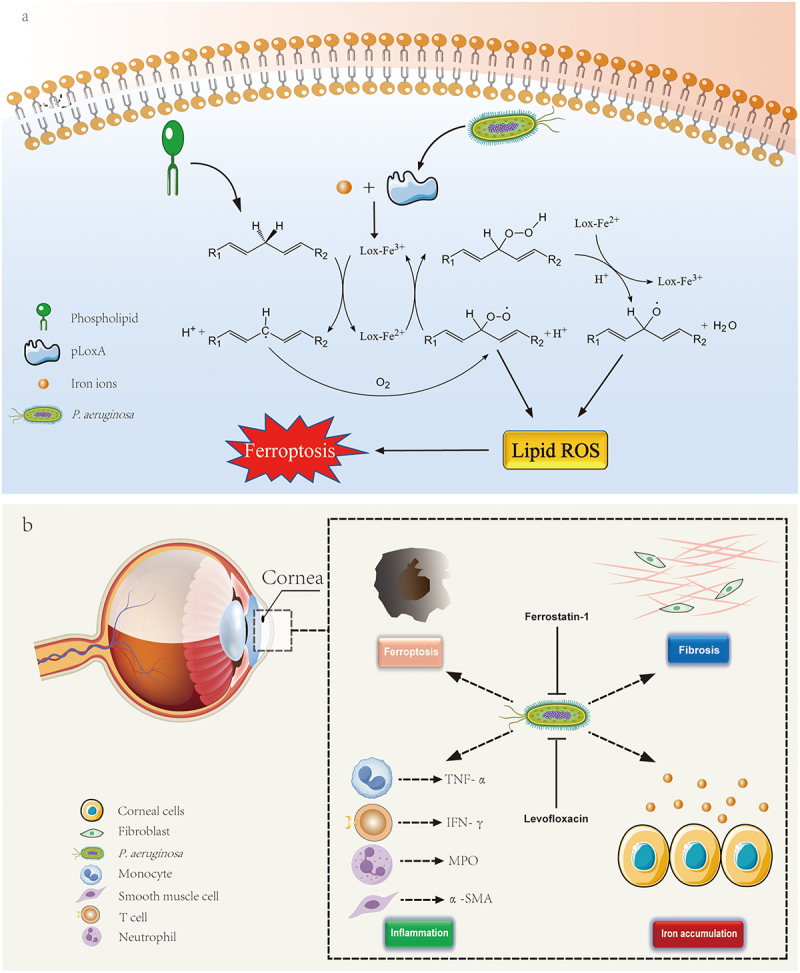
Diseases associated with ferroptosis caused by *P. aeruginosa*. a) within respiratory epithelial cells, *P. aeruginosa* secretes its own lipoxygenase pLoxa, that facilitates the conversion of polyunsaturated fatty acids into ROS, thereby inducing ferroptosis. b) in bacterial keratitis, *P. aeruginosa* can promote the accumulation of iron ions, causing corneal cells to undergo ferroptosis, while also triggering inflammation and fibrosis of corneal tissue.

Moreover, *P. aeruginosa* can degrade the important anti-ferroptotic enzyme glutathione peroxidase 4 (GPX4) in host cells through the chaperone-mediated autophagy (CMA) pathway, leading to a reduction in the key antioxidant glutathione (GSH) [52].

Furthermore, siderophores promote the production of free radicals in host cells, leading to tissue damage. Studies have demonstrated that certain *P. aeruginosa* strains carrying compensatory mutations in the *rne* gene (encoding RNase E) can parasitize macrophages, upregulate siderophore expression on their surface, and disrupt intracellular iron homeostasis. Iron-bound pyochelin has been shown to induce macrophage ferroptosis and subsequent lysis in this process [20]. Additionally, pyochelin and iron have been found to catalyze hydroxyl radical production through the Haber-Weiss reaction [81]. Pyoverdine, which exhibits higher iron-binding affinity, can extract iron from human transferrin and plays a crucial role in *P. aeruginosa* acute infections [82], and production of two important virulence factors, exotoxin A and PrpL protease, is regulated by it [24]. Another important virulence factor, pyocyanin, functions as both a redox-active secondary metabolite and a quorum-sensing (QS) signaling molecule during *P. aeruginosa* infections [83].

Currently, *P. aeruginosa* has been found to elevate lipid peroxidation levels and ROS in bronchial epithelial cells and pulmonary cells in various diseases, including acute lung injury, acute respiratory distress syndrome, bacterial keratitis, and cystic fibrosis [6,84,85]. In the respiratory tract, *P. aeruginosa* induces peroxidation of membranes through the previously mentioned lipoxygenase mechanism, leading to extensive ferroptosis in host cells. In bacterial keratitis, *P. aeruginosa* reduces GPX4 and SLC7A11 in host cells while promoting iron accumulation in tissues, causing ferroptosis in the cornea. This is accompanied by upregulation of pro-inflammatory and repair-related molecules such as IFN-γ, TNF-α, MPO, and α-SMA, which contribute to inflammation and fibrosis [84] (Figure 1(b)). Additionally, previous studies have found that the induction of ferroptosis can inhibit the proliferation, migration, invasion, and survival of various cancer cells [86]. For example, progesterone enhances the effect of niraparib in inducing DNA damage and death in ovarian cancer cells by upregulating palmitoleic acid to induce ferroptosis and cause mitochondrial damage; allicin can induce ferroptosis in human nasopharyngeal carcinoma cells by reducing the levels of GSH and GPX4 and promoting the formation of toxic LPO and ROS [87]. However, *P. aeruginosa* in tumor microenvironments secretes pyoverdine to sequester extracellular iron ions, thereby reducing intracellular iron availability and diminishing tumor cell susceptibility to ferroptosis (Figure 1(c)). Additionally, *P. aeruginosa* downregulates transferrin receptor expression in tumor cells, thereby decreasing ROS production. Furthermore, the presence of *P. aeruginosa* and other tumor-associated microbes can promote the epithelial–mesenchymal transition in tumor tissues and increase the expression of invasion-related molecules such as CD44, CD133, and CD166 [32].

**Figure 1.**
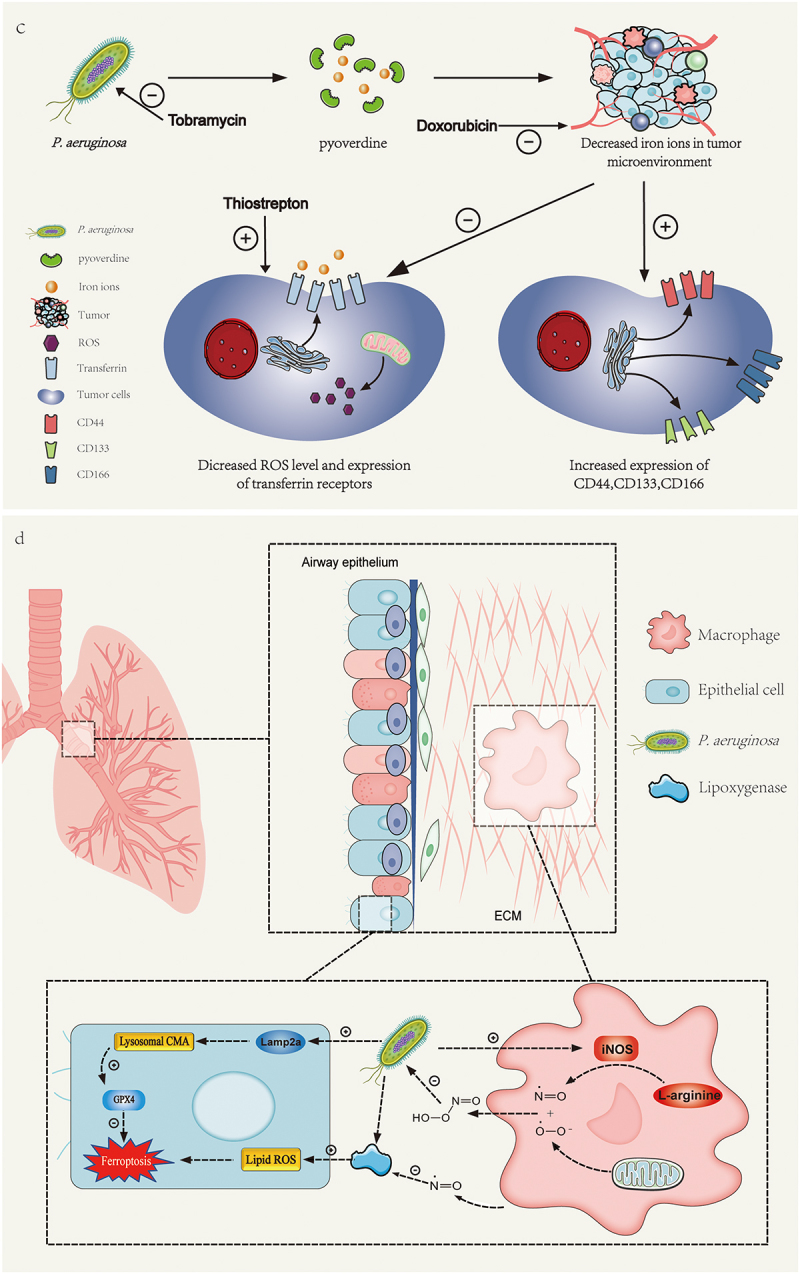
c) *P. aeruginosa* can acquire iron ions from the tumor microenvironment through pyoverdine, which decreases the level of ROS in tumor cells, thus reducing the incidence of ferroptosis. Concurrently, this process enhances the expression of tumor invasion-related molecules such as CD44, CD133, and CD166. Applying tobramycin, ferroptosis-inducing thiostrepton, and anti-cancer doxorubicin could eradicate biofilms and tumors. d) in airway epithelium, P. aeruginosa stimulates the expression of Lamp2a, a crucial molecule in the chaperone-mediated autophagy pathway within epithelial cells, leading to the degradation of GPX4 in the antioxidant system. However, adjacent macrophages can activate iNOS to produce nitric oxide, which not only kills the pathogen but also diffuses into epithelial cells to exert a protective effect.

### Host resistance to ferroptosis caused by *P. aeruginosa*

Ferroptosis not only leads to host cell death but may also activate the host immune response through the release of damage-associated molecular patterns (DAMPs). However, *P. aeruginosa* may evade immune clearance by suppressing ferroptosis-related signaling pathways. For example, research has revealed that host cells induce the production of inducible nitric oxide synthase (iNOS)/NO, initiating a ferroptosis-resistant mechanism that operates independently of the GPX4 antioxidant pathway. This mechanism inhibits excessive lipid peroxidation, protects cells deficient in GPX4 and GSH, and controls cellular ferroptosis [52]. Studies have demonstrated that nitric oxide (NO), as a small molecular signaling molecule and a product of iNOS, can deactivate iron-containing enzymes by binding to them or react with superoxide anion radicals (O_2_^•−^) produced in the mitochondria to form highly reactive peroxynitrite (OONO^−^), which directly attacks pathogens (Figure 1(d)). NO can propagate between cells and exert an anti-ferroptotic effect, positioning it as a potential therapeutic target, especially in immunocompromised patients. Moreover, the mechanism of action of NO is independent of GPX4, providing new insights into how host cells resist cell death.

## Potential iron-based therapeutic strategies

### Therapies targeting on bacterial iron acquisition and metabolism

**Figure 2.**
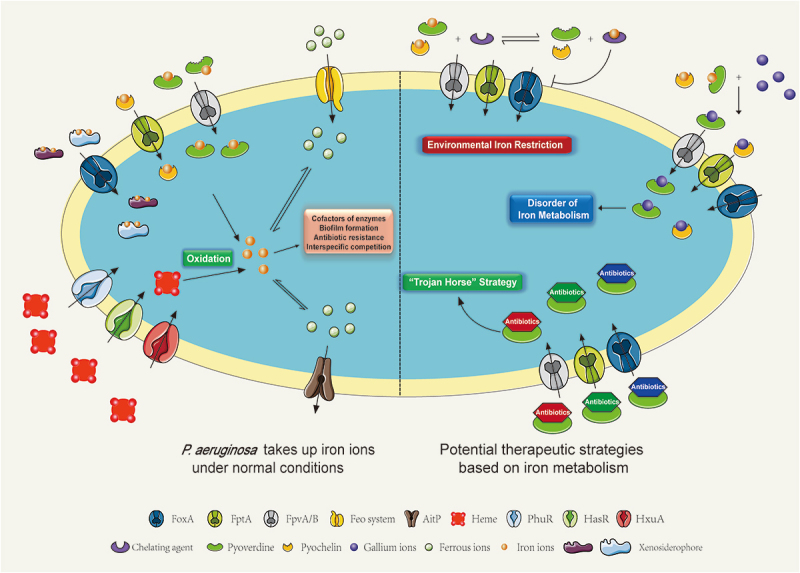
Uptake and metabolism of irons in *P. aeruginosa* and potential therapeutic targets. Left: *P. aeruginosa* acquires iron ions necessary for survival by secreting siderophores, utilizing heme from host cells, and taking up xenosiderophores from other species. The iron is internalized and metabolized through siderophore receptors such as FoxA, FpvB, and FiuA. Right: potential therapeutic targets include: (1) creating environmental iron limitation by using iron chelators to sequester iron; (2) disrupting iron metabolism with gallium ions, which have similar chemical properties to iron ions; and (3) employing a “Trojan horse” strategy to deliver antibiotics into the bacterial cells by conjugating them with siderophores.

Therapeutic and pharmacological research targeting the iron metabolism pathways of *P. aeruginosa* has been extensively conducted, with three primary strategic approaches emerging: (1) Using iron chelators to restrict bacterial uptake of iron from the environment; (2) Exploiting the bacterial iron uptake mechanism to deliver drugs into the cell; (3) Using metal ions to mimic iron ions and interfere with bacterial metabolism (Figure 2) [19].

Iron chelators attenuate *P. aeruginosa* virulence by sequestering environmental iron, thereby establishing iron-restricted conditions. Substantial experimental evidence confirms the efficacy of this approach. Notably, coumarin derivatives and polyvinylpyrrolidone (PVP) function as high-affinity iron chelators that effectively compete for iron ions, inducing iron starvation in *P. aeruginosa* [88]. A separate study demonstrated that FDA-approved iron chelator deferoxamine and deferasirox, when combined with tobramycin, reduced existing biofilm biomass by approximately 90% and decreased viable bacterial counts by 7 log units. These findings suggest their potential as an effective therapeutic strategy for cystic fibrosis (CF) patients and other pulmonary conditions involving antibiotic-resistant *P. aeruginosa* biofilms [89]. Additionally, deferiprone (DFP)-loaded layered double hydroxide (LDH)-based nanomedicine (DFP@Ga-LDH), as a novel therapeutic material, can alter the Fe/Ga ratio in *P. aeruginosa* during treatment of hematoma-induced implant-associated infections. This modification induces broad-spectrum interference with multiple iron-related pathways, including transcriptional regulation and metabolic processes, while simultaneously delaying the development of drug resistance [90].

In addition to reducing the concentration of iron ions in the environment, “Trojan horse” strategy, which involves targeting drugs to the bacterial interior via siderophores, can be used to deliver antibiotics, thereby bypassing the bacterial cell membrane defenses [91]. Siderophores currently designed include natural pyoverdine and synthetic carriers (such as catechol-based compounds). Studies have shown that these carriers can deliver antibiotics, such as fluoroquinolones, thiostrepton and β-lactam antibiotics, into *P. aeruginosa* through various TonB-dependent transporters or the outer membrane receptor FptA or FpvB of siderophores, helping to restore antibiotic sensitivity in drug-resistant *P. aeruginosa* [17,92,93]. In addition, studies have also shown that catechol-conjugated curcumin can achieve inhibitory effects against *P. aeruginosa* [94].

Gallium ions, developed as candidates for antibacterial therapy due to their chemical similarity to iron ions, inhibit bacterial growth by mimicking iron and disrupting iron metabolism [95]. Several gallium compounds have been proven effective, including gallium nitrate, gallium maltolate (GaM), and desferrioxamine-gallium (DFO-Ga). Different Ga^3 +^ formulations exhibit distinct effects after entering cells, suggesting multiple targets [41]. Gallium ions inhibit the growth and biofilm formation of *P. aeruginosa* by reducing bacterial iron uptake and interfering with iron signaling mediated by the transcriptional regulator pvdS [16]. Additionally, gallium can be combined with sodium nitrite to disrupt iron metabolism and quorum sensing systems [96]. GaM significantly upregulates the expression of proteins related to iron acquisition and storage while reduces the abundance of proteins related to quorum sensing and chemotaxis [97]. DFO-Ga, leveraging the “Trojan horse” strategy mentioned earlier, exhibits stronger toxicity than free gallium ions and significantly enhances the bactericidal effect when combined with the antibiotic gentamicin [98,99].

### Therapies targeting on ferroptosis mechanism

Given that ferroptosis is a crucial mechanism underlying *P. aeruginosa*-induced disease and cell death, it is necessary to explore the inhibition of ferroptosis as a therapeutic strategy for *P. aeruginosa* infections. The hallmark events of ferroptosis include the intracellular accumulation of iron and reactive oxygen species (ROS), inhibition of system Xc^−^, depletion of glutathione, oxidation of nicotinamide adenine dinucleotide phosphate (NADPH), and lipid peroxidation. Targeting these features for inhibition could represent potential therapeutic targets for treating *P. aeruginosa* infections [100].

Ferroptosis inhibitors like ferrostatin-1 (Fer-1), liproxstatin-1, α-tocopherol, FSP1, Coenzyme Q10, and BH4 are capable of blocking the lipid peroxidation cascade and demonstrated significant therapeutic effects when used in combination with other drugs [101]. The combined use of levofloxacin and the ferroptosis inhibitor Fer-1 has been proven to reduce inflammation levels in bacterial keratitis while restoring ROS, ferrous ions, GPX4, and SLC7A11, and other molecules related to ferroptosis in corneal stromal stem cells to normal levels [84]. Another study indicated that the application of antioxidants (coenzyme Q10) and the ferroptosis inhibitor Fer-1 also reduced lipid peroxidation levels in mice with cystic fibrosis, presenting another potential therapeutic approach [85]. Liproxstatin-1 and MAC-0568743, a novel cationic amphiphile, were shown to compromise outer membrane integrity through specific interactions with lipopolysaccharide in Gram-negative bacteria [102].

In addition, the lipoxygenase unique to *P. aeruginosa* is highly conserved evolutionarily. Inhibiting this enzyme, which initiates lipid peroxidation and ferroptosis, may represent a potential therapeutic target for *P. aeruginosa*-related diseases, such as cystic fibrosis (CF) and chronic lower respiratory tract infections [78].

### Antibacterial properties of high iron ions concentration

As mentioned earlier, although iron ions play an essential role in maintaining the pathogenicity and normal life activities of P. aeruginosa, excessively high levels of iron ions can promote the production of ROS, leading to bacterial death, presenting another viable antibacterial strategy. Many iron-containing small-molecule agents can induce a ferroptosis-like mechanism in bacteria. Currently, ferric chloride, ferrous sulfate and chloride (N,N’-bis(salicylidene)-1,2-phenylenediamine iron(III) chloride have all been reported to induce ferroptosis-like mechanisms in both Gram-positive and Gram-negative bacteria, demonstrating significant antibacterial properties [14,103,104].

**Figure 3.**
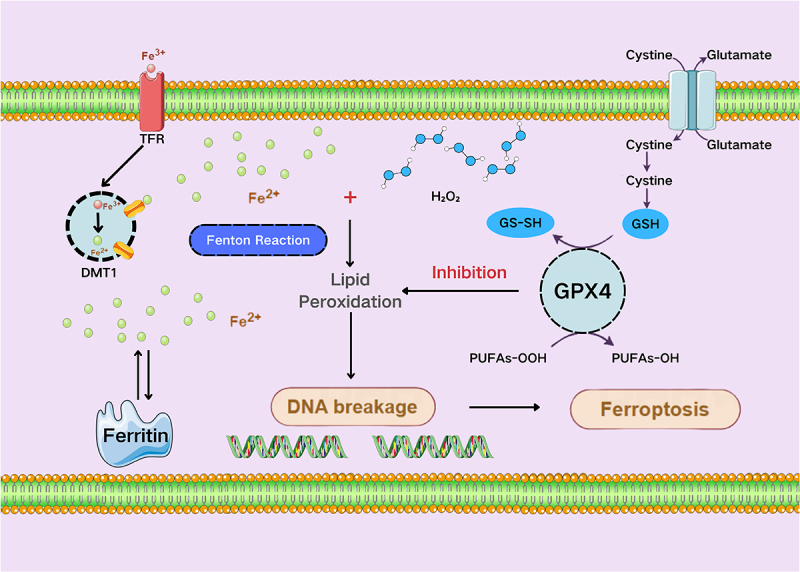
The mechanisms of ferroptosis in *P. aeruginosa*. Ferroptosis in *P. aeruginosa* can be induced by excessive iron ions. The inhibition of GPX4, is the key step of ferroptosis, which in turn leads to an overabundance of intracellular ROS and lipid peroxidation through the Fenton reaction, damaging cellular proteins and DNA, leading to cell death. PUFA, polyunsaturated fatty acyl moieties; TFR, transferrin receptor; DMT1, divalent metal transporter 1.

Previous studies in animal experiments have been reported that excessive iron compounds induce *P. aeruginosa* ferroptosis, aiding skin burn wound healing; 200 µM ferric chloride carried by thermal-responsive hydrogels, generates ROS that causes oxidative stress and lipid peroxidation, leading to DNA breakage, killed more than 99.9% of *P. aeruginosa* cells [14] (Figure 3).

Both Fe^3 +^ and Fe^2 +^ have been demonstrated to exert potent antibacterial effects through ferroptosis-like mechanisms in *P. aeruginosa*. Ferrous sulfate treatment induces multiple physiological disruptions, including reduction of the glutathione/oxidized glutathione (GSH/GSSG) ratio accompanied by downregulation of γ-glutamyltransferase (*ggt*) gene expression, intracellular iron overload indicated by upregulated ferrochelatase (hemH), and elevated reactive oxygen species (ROS) levels accompanied by lipid peroxidation. Addionally, the treatment disrupts cell membrane integrity and inhibits *P. aeruginosa* biofilm formation on diverse substrates through downregulation of key biofilm-related genes (*ppkA*, *chpC*, *algA*, and *alg44*) [103].

In addition, a study has demonstrated that extracellular vesicles secreted by *P. aeruginosa* during its exponential growth phase can promote the growth of biofilms, while extracellular vesicles secreted during the death/survival phase can effectively inhibit or eliminate the biofilm of *P. aeruginosa* (D-EVs). Furthermore, the inhibitory effect of D-EVs on biofilms is further enhanced in the presence of 10–50 μM Fe^3+^ ions. Their proteomic analysis suggests that this inhibitory effect involves an iron-dependent ferroptosis mechanism, and D-EVs may play a key role in bacterial programmed cell death by promoting iron uptake and inducing the production of ROS [105].

However, it should be mentioned that although ferroptosis has been confirmed to occur in *Aspergillus flavus*, *Staphylococcus aureus*, *Vibrio parahaemolyticus*, and *P. aeruginosa*, and effectively inhibits their reproduction [106–108], however, due to the fact that the phospholipids on the surface of bacteria are mostly composed of saturated fatty acids or monounsaturated fatty acids, inducing ferroptosis in bacteria is more difficult than inducing mammalian cells [109,110]. Moreover, since the Unsaturated Iron Binding Capacity in healthy individuals (12–43 µmol/L in males; 13–56 µmol/L in females) is in the same order of magnitude as the concentration of iron ions added in the literature (total amount of 200 µmol), it is debatable whether excessive iron ions, while killing *P. aeruginosa*, might also cause damage to tissue cells [111].

In conclusion, the induction of ferroptosis in *P. aeruginosa* presents a promising yet complex target for therapeutic intervention. By elucidating the molecular pathways, future research may pave the way for innovative treatments that mitigate the detrimental effects of this opportunistic pathogen.

## Conclusion and prospectives

This review underscores the critical role of iron metabolism in the survival, growth, and pathogenicity of *P. aeruginosa*. The complex iron acquisition systems of the bacterium, including siderophore production and heme utilization, not only constitute essential metabolic enzymes vital for its life activities but also enhance its virulence, promote biofilm synthesis, and play a significant role in interspecies competition. However, iron can be both a nutrient and a weapon. On one hand, iron is crucial for bacterial growth; on the other hand, it can be used to trigger host cell death, thereby facilitating bacterial dissemination. Conversely, high iron environments have been shown to induce ferroptosis in *P. aeruginosa*, offering a potential therapeutic strategy against this pathogen.

The intricate interactions between *P. aeruginosa* and its host, mediated in part by iron metabolism, highlight the need for innovative approaches to disrupt this relationship. The development of therapies targeting iron acquisition mechanisms, such as siderophore sequestration, interference with siderophore synthesis regulation, inhibition of iron transport proteins, using metal ions with similar chemical properties to iron ions to disrupt iron metabolism, and employing a “Trojan horse” strategy to deliver drugs into bacterial cells via siderophores, may provide new avenues for combating drug-resistant strains [112,113]. Additionally, understanding the molecular mechanisms underlying bacterial ferroptosis presents an opportunity to exploit this pathway for therapeutic benefit.

Looking ahead, research should focus on elucidating the precise mechanisms by which *P. aeruginosa* acquires iron and disrupts host cell iron homeostasis, thereby inducing lipid peroxidation and downregulating the antioxidant system. Identifying specific targets within the iron metabolism pathways and the application of ferroptosis inhibitors could lead to the development of therapies that inhibit bacterial growth through mechanisms different from traditional antibiotics.

The potential of using iron compounds to induce bacterial ferroptosis warrants further exploration [114]. Understanding the differences in ferroptosis mechanisms between prokaryotes and eukaryotes could reveal new vulnerabilities in *P. aeruginosa* that can be exploited therapeutically. Moreover, the study of extracellular vesicles and their role in mediating bacterial ferroptosis represents an exciting frontier in microbiology. However, it is also essential to fully consider the impact of high concentrations of iron ions on host tissue cells and to develop new strategies that inhibit bacterial growth while minimizing damage to tissue cells.

However, therapeutic strategies targeting iron metabolism in *P. aeruginosa* must carefully consider potential adverse effects on host cells and tissues. Whether employing iron chelators to inhibit bacterial iron uptake, gallium ions to disrupt iron metabolism, or elevated iron concentrations to induce bacterial ferroptosis, these interventions require careful evaluation of their systemic impacts. Maintaining iron homeostasis is crucial for human physiology, as both iron overload and deficiency can lead to multisystem dysfunction. In conditions of iron overload, excess free iron catalyzes reactive oxygen species (ROS) generation through Fenton reactions, resulting in oxidative damage. This pathogenic mechanism is particularly evident in diabetes mellitus, where it directly impairs pancreatic β-cell function and exacerbates vascular complications including atherosclerosis and retinopathy [86,115]. Furthermore, iron overload is associated with increased risks of liver fibrosis and neurodegenerative disorders like Alzheimer’s disease [116,117]. Conversely, iron deficiency impairs hemoglobin synthesis, causing iron-deficiency anemia with manifestations such as fatigue and cognitive decline. Metabolically, iron deficiency disrupts mitochondrial respiratory chain enzymes, compromising energy production [118]. Notably, iron imbalance also affects other metal ions: iron deficiency may induce intracellular calcium dysregulation [119], while chronic deficiency correlates with immune impairment and delayed wound healing [120].

In conclusion, the battle against *P. aeruginosa* requires a multifaceted approach that incorporates a deep understanding of its iron metabolism and host – pathogen interactions. By targeting bacterial iron acquisition and manipulation of ferroptosis pathways, we may be able to develop more effective treatments against this formidable pathogen, ultimately improving patient outcomes and curbing the spread of antibiotic resistance.

## Data Availability

Data sharing isn’t applicable to this article as no new data were created or analyzed in this study.
